# Chronic Cerebrospinal Venous Insufficiency: Case–Control Neurosonography Results

**DOI:** 10.1002/ana.23839

**Published:** 2013-02-26

**Authors:** Andrew D Barreto, Staley A Brod, Thanh-Tung Bui, James R Jemelka, Larry A Kramer, Kelly Ton, Alan M Cohen, John W Lindsey, Flavia Nelson, Ponnada A Narayana, Jerry S Wolinsky

**Affiliations:** 1 Department of Neurology, University of Texas Health Science Center at HoustonHouston, TX; 2 Departments of Diagnostic and Interventional Imaging, University of Texas Health Science Center at HoustonHouston, TX

## Abstract

**Objective:**

Chronic cerebrospinal venous insufficiency (CCSVI) has been implicated in the pathophysiology of multiple sclerosis (MS). We sought to determine whether neurosonography (NS) provides reliable information on cerebral venous outflow patterns specific to MS.

**Methods:**

This was a single-center, prospective case–control study of volunteer MS and non-MS participants. A neurosonologist, blind to the subjects' diagnosis, used high-resolution B-mode imaging with color and spectral Doppler to systematically investigate, capture, and record extracranial and intracranial venous drainage. These neuroimaging results were evaluated and scored by an expert blinded to subjects' information and with no interactions with the participants.

**Results:**

Altogether, 276 subjects were studied: 206 with MS and 70 non-MS. MS patients were older than non-MS subjects (48.3±9.9 vs 44.3±11.8 years, *p*<0.007), with durations from first symptoms and diagnosis of 13.7±10 and 9.9±7.8 years, and Expanded Disability Status Scale of 2.6±2.0. Overall, 82 subjects (29.7%) fulfilled 1 of 5 NS criteria proposed for CCSVI; 13 (4.7%) fulfilled 2 criteria required for diagnosis, and none fulfilled >2 criteria. The distribution of subjects with 0, 1, or 2 criteria did not differ significantly across all diagnostic groupings, between MS and non-MS subjects, or within MS subgroups. CCSVI was present in 7.14% of non-MS and 3.88% of MS patients (*p*=0.266). No significant differences emerged between MS and non-MS subjects for extracranial or intracranial venous flow rates.

**Interpretation:**

NS findings described as CCSVI are much less prevalent than initially reported, and do not distinguish MS from other subjects. Our findings do not support the hypothesis that CCSVI is causally associated with MS. ANN NEUROL 2013;73:721–728

Multiple sclerosis (MS) is generally accepted as an immune-mediated inflammatory disease triggered by unknown factor(s). However, this pathophysiology has been challenged recently by Zamboni and colleagues, who have described a new disorder called chronic cerebrospinal venous insufficiency (CCVSI). Initially defined by the presence of 2 or more disordered venous outflow parameters as measured by intra- and extracranial duplex ultrasound, CCSVI was originally reported to have 100% overlap with the diagnosis of MS, but was not encountered in other diseases or normal controls.^1^,^2^ These investigators theorized that insufficient venous drainage resulted in iron accumulation and enhanced central nervous system inflammation.

Following the original publications, independent investigators well known for their ultrasound expertise have tried to duplicate the findings. Doepp et al evaluated 56 patients with MS and 20 controls in a case–control study.^3^ No jugular stenoses were found and the blood flow direction was normal in all but 1 of their subjects. None of their MS patients fulfilled >1 criterion for CCSVI. Baracchini and colleagues failed to find a cause–effect relationship between CCSVI and clinically isolated syndromes or progressive MS.^4^,^5^ Tsivgoulis et al studied 42 MS patients and 43 non-MS controls. Two sonographers, blinded to diagnosis, performed the CCSVI protocol and found 1 MS (2%) and 1 control (2%) had reflux in the internal jugular vein during apnea.^6^ None of the participants met criteria for CCSVI (≥2 ultrasound abnormalities).

The largest study published to date (499 participants including 289 patients with MS) by Zivadinov et al found that 56% of patients with MS met ultrasound criteria for CCSVI as did 23% of healthy controls.^7^ Of note, the neurosonologist who performed the ultrasound procedures in this study was trained directly in the University of Ferrara laboratory. Zivadinov and colleagues concluded that CCSVI was unlikely to have a primary causative role in MS.

Despite lack of reproducibility among investigators, patients with MS have undergone endovascular balloon and stent venoplasty procedures to correct venous abnormalities. In a few cases, patients have been harmed, with injuries ranging from migrated jugular stents to fatal brainstem intracerebral hemorrhages.^8^

The primary purpose of this portion of our study was to compare the prevalence of CCSVI as defined by neurosonography (NS) in MS and non-MS subjects. We also sought to evaluate what other diagnostic approaches might be the best to determine whether altered venous outflow is associated with MS. All subjects underwent evaluation by NS, and a subset of MS subjects were invited to undergo magnetic resonance (MR) venography and transluminal venography. Here we present the results of the neurosonographic portion of the study.

## Subjects and Methods

This was a single-center, prospective, case–control study that enrolled MS and non-MS volunteers at the University of Texas Health Science Center at Houston. MS patients were recruited from the MS program of the university neurology clinic and were aged 18 to 65 years, without a history of venoplasty. Non-MS subjects included healthy controls recruited from employees at the university and individuals with other neurological diseases invited from the general and vascular disease specialty programs of the university neurology clinic. Volunteers met inclusion criteria if were ≤65 years old (1 subject was inadvertently entered at age 67 years), had no history of venous disease (eg, cerebral venous thrombosis), had no history of intracerebral hemorrhage within 6 months, had no right-sided congestive heart failure, had no history of internal jugular vein (IJV) cannulation, and were without a history of venoplasty. All participants needed to be able to transition from a seated to supine position without assistance. The study was approved by the institution's Committee for the Protection of Human Subjects and performed in an ICAVL (Intersocietal Commission for the Accreditation of Vascular Laboratories) accredited neurosonologic facility.

### Neurosonography Protocol

A certified neurosonologist (T.-T.B.), blind to the subject's diagnosis, used high-resolution B-mode imaging with color and spectral Doppler to investigate the venous drainage (Philips CX50; Philips Medical Systems, Bothel, WA), consistent with the Zamboni publication.^2^ Extracranial vessels were studied using a phased linear array transducer (12–3 MHz), and intracranial vessels were probed using a phased sector array transducer (5–1MHz). The system was optimized for venous imaging by using a low wall filter and low pulse repetition frequency (PRF).

### Extracranial Vessels: Internal Jugular and Vertebral Veins

Using the B-mode setting in the supine position, both IJVs were surveyed in real time from the base of the neck, and the probe was moved slowly cephalad to the mastoid process in the transverse plane. A slight compression of the vein was performed to check venous patency or for the presence of thrombosis. The presence of IJV valves (or any anomalies), usually located near the confluence of the brachiocephalic vein, was documented, and both still images and cine-loop video were recorded. Any abnormalities in either jugular were recorded. The cross-sectional area (CSA) was reviewed in grayscale and color, but documented in grayscale in a transverse view at the level of the mid thyroid gland (or at the level of the smallest CSA) during the expiratory phase and with only slight probe pressure to avoid vein compression. According to Zamboni criterion #3, the jugular was judged to be stenotic if the CSA was ≤50% and/or ≤0.3cm^2^.^1^,^2^

**FIGURE 1:**
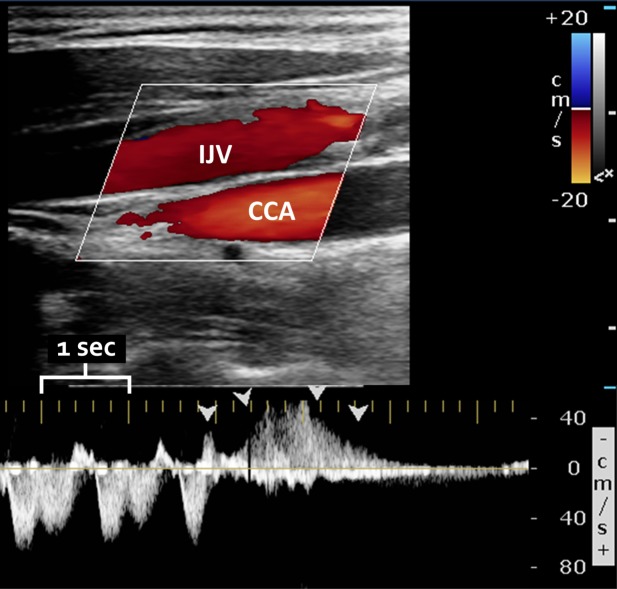
Sample color (top) and spectral (bottom) Doppler ultrasound of the internal jugular vein (IJV), demonstrating reflux (with the same direction of flow as the underlying common carotid artery [CCA]). The large yellow tick marks indicate 1-second intervals. Arrowheads demonstrate reflux lasting >0.88 seconds. Abnormal reflux was only determined using the spectral Doppler waveform, which allows precise determination of flow direction and duration.

The jugular CSA was repeated at the same anatomic level in an upright position (90°) and subtracted from the supine value. If the upright CSA was greater than the supine in either jugular, the subject was deemed abnormal for this parameter (Zamboni criterion #5, negative change in the CSA in the jugular vein). Angle-corrected spectral Doppler velocities (cm/s; maximal and minimal velocities in a similar fashion to peak systolic and end diastolic arterial studies) and waveforms in the sagittal plane were generated for both vertebral and jugular veins at 0 and 90°. The PRF was adjusted to optimize the waveform, and the sample gate was set to 1.8 to 3.4 mm (depending on vessel size) and always placed in the center of the vessel according to standard vascular procedures. Spectral waveforms were performed in real time as the patient was instructed to breathe normally. After 5 seconds of normal breathing, the patient was asked to hold their breath (apnea) for 5 seconds following a normal exhalation, being careful not to perform a Valsalva maneuver. After 5 seconds of apnea, the patient was asked to again breathe normally, being careful not to forcefully exhale/inhale, as the spectral gate can be displaced from the vein by excessive movement. Venous reflux of flow was solely determined using spectral Doppler waveforms (not color Doppler; Fig 1) and defined as a flow reversal of >0.88 seconds duration and ≥12 cm/s velocity in either position (after apnea) in any jugular or vertebral vein (Zamboni criterion #1).^9^ In addition to during apnea, the same vessels were also evaluated during a Valsalva maneuver using a tabletop Valsalva meter (American Diagnostics, Hauppauge, NY). The patient was instructed to breathe normally for 10 seconds, then perform a Valsalva maneuver and hold the meter at 40 mmHg for 10 seconds. Valsalva-induced reflux was not used in the calculation of the Zamboni score. When either the jugular or vertebral veins' walls were clearly demonstrated on B-mode imaging, but no color or spectral Doppler signal could be obtained despite image optimization efforts (increasing the color/gain or lowering the scale), the patient was classified as having “flow not Doppler detectable” (Zamboni criterion #4).

### Intracranial Vessels: Deep Cerebral Veins

Using a transtemporal approach, the deep cerebral veins (basal vein of Rosenthal, internal cerebral veins, and great vein of Galen) were imaged. First, the hypoechoic midbrain was visualized using grayscale imaging. Next, a color box was placed over and lateral to the midbrain to image the posterior cerebral artery (PCA) color flow. The basal vein of Rosenthal is routinely located lateral to the PCA and was confirmed when a venous spectral Doppler waveform was produced. Finally, a spectral Doppler waveform was produced with a sample gate of 1.8 mm. The patient was instructed to breathe normally, and in addition the Valsalva maneuver was also performed as described above. Similar techniques were used to visualize and study other deep cerebral veins. Intracranial reflux was deemed abnormal if the duration on spectral Doppler was >0.5 seconds (Zamboni criterion #2).

### Blinding

Neurosonography was performed in a blinded fashion. The neurosonologist (T.-T.B.) had no access to any subject's demographic characteristics or diagnosis. Study participants and the neurosonologist were instructed not to discuss their medical history at any time before, during, or after the examination. All digital neurosonology images and velocity data were saved at the end of each day to a secure server. These were subsequently independently evaluated by A.D.B., who was blind to any subject information and had no contact with study participants. Only after all subjects were recruited and all of their ultrasound and any other subsequent vascular investigations and imaging interpretations were completed, and the database was locked, were linked data seen by any study investigators other than J.S.W. Subjects who underwent NS were invited to pass through successive phases of testing based on their results and evolving results in the assembled cohort of subjects, and the need to have examples of subjects both with and without demonstrated abnormalities at each subsequent level of investigation. Although not reported further here, additional test levels included MR venography (MRV) and transluminal venography (TLV). These were performed by an MR technologist and an experienced venographer, respectively, both unaware of the other's results, of the NS testing, and of the subject's diagnosis. Only after the interpretation of all NS, MRV, and TLV was completed, all queries of the data were made, and the database was locked, were any discussions of the results allowed among the experts at the level of individual subjects. This was done to preserve the blinded and independent evaluation of each test. Only then was the entire team allowed to determine the consistency of results across the major investigative tests.

### Sample Size and Statistical Analysis

We originally envisioned recruiting 100 MS subjects and 175 non-MS control subjects. The non-MS control subjects were to be drawn from several different patient pools: ∼100 subjects undergoing evaluation for cerebral vascular disease by our stroke group, ∼75 subjects under evaluation or management by our diagnostic neurology group, and ∼10 healthy volunteers. Based on the original publication,^2^ it was assumed that this would provide ample power to confirm or question the specificity of the proposed NS criteria for the diagnosis of CCSVI. Specifically, we projected >99% power to find a 50% difference in the prevalence of CCSVI among 100 MS subjects compared to the universal presence of CCSVI among the 109 MS subject cohort used by the Ferraro group (at 2-sided alpha=0.05). However, shortly after our 12-month project update, when the proportion of all of our subjects with NS findings consistent with CCSVI was 0.07 and similar between the MS and non-MS volunteers, J.S.W., in consultation with our local Executive Committee and National MS Society staff, modified our subject recruitment plan to be consistent with both the evolving data and difficulty encountered in the timely recruitment of non-MS subjects. Shifting the recruitment emphasis allowed us to recruit greater numbers of MS subjects while continuing to recruit non-MS subjects; the shift in emphasis was not discussed with those members of the team involved in direct testing of any subjects in the study.

Pearson chi-square test (Fischer exact test where appropriate) and unpaired *t* test were used for comparisons of categorical and continuous variables, respectively, between MS and non-MS subjects; continuous variables were subjected to 1-way analyses of variance. A 2-tailed *p* value of <0.05 was considered statistically significant. The statistical package JMP 10.0.1 (release 2; SAS Institute, Cary, NC) was used for analyses.

## Results

From June 2010 through March 2012, 276 blinded NS evaluations were completed. Of these, 206 were MS patients, and 70 were non-MS (Table 1). Patients were older than non-MS subjects (48.3±9.9 vs 44.3±11.8 years, *p*<0.007), but the proportion of females was not different (71.4 vs 64.3%, *p*=0.266). For MS patients, the duration of symptoms (years±standard deviation [SD]), duration of diagnosis (years±SD), Expanded Disability Status Scale (mean±SD), and percentage not receiving disease-modifying therapies were 13.7±10, 9.9±7.8, 2.6±2.0, and 20%, respectively. The number of MS subjects by clinical disease phenotype is provided in Table 1. Further subgroup demographic data are available as Supplementary Table 1. At least 1 deep cerebral vein was successfully imaged in 264 of 276 (95.6%) and at least 2 veins in 241 of 276 (87.3%) subjects. Therefore, only 12 of 276 (4.3%) lacked temporal windows adequate for intracranial duplex imaging.

**TABLE 1:** Study Participant Demographics

Participants	Number
MS	206

Relapsing–remitting	128

Secondary progressive	48

Primary progressive	15

Clinically isolated syndrome	12

Progressive relapsing	3

Non-MS	70

Other neurological diseases	37

Headache	22

Neuropathy	3

Trigeminal nerve disorders	3

Disk disease and neck pain	3

Encephalitis	2

Alzheimer dementia	1

Brachial neuritis	1

Neuromyelitis optica	1

Brain tumor	1

Cerebrovascular diseases	22

Cerebral infarction	17

Stroke syndrome	1

Carotid artery occlusion	1

Intracerebral hemorrhage	1

Generalized convulsion	1

Carotidynia	1

Healthy controls	11

MS=multiple sclerosis.

Eighty-two of the 276 subjects fulfilled at least 1 of the Zamboni criteria (29.7%), and 13 had 2 criteria consistent with the definition of CCSVI (4.7%). Tables 2 and 3 provide NS assessment results by subject and the number of criteria met. No patients were found to have reflux of the deep cerebral veins or >2 criteria. The distribution of subjects with 0, 1, or 2 criteria did not differ significantly across all diagnostic groupings, between MS and non-MS subjects, or within the MS subgroups. The proportion of MS (3.88%) and non-MS (7.14%) subjects with at least 2 criteria did not differ (*p*=0.266, Pearson chi-square).

**TABLE 2:** Neurosonography Results Tabulated by Participant Category

Diagnosis	All Subjects	Subjects at Zamboni Criteria		
		
		0	1	2
MS, clinically isolated syndrome	12	8	4	0

MS, relapsing–remitting	128	84	41	3

MS, secondary progressive	48	29	15	4

MS, primary progressive	15	9	5	1

MS, progressive relapsing	3	3	0	0

Healthy volunteers	11	9	2	0

Other neurologic diseases	37	26	7	4

Stroke/TIA	22	13	8	1

All	276	181	82	13

MS=multiple sclerosis; TIA=transient ischemic attack.

**TABLE 3:** Neurosonography Results Comparing the 5 Chronic Cerebrospinal Venous Insufficiency Criteria across MS versus Non-MS Participants

Zamboni Criteria	Subjects with Zamboni Criteria, %			
	
	1		2	
		
	MS	Non-MS	MS	Non-MS
1. Extracranial reflux	1.9	0	0.5	1.4

2. Intracranial reflux	0	0	0	0

3. B-mode jugular stenosis	25	20	3.9	5.7

4. No flow detected on Doppler	4.4	2.9	1.5	2.9

5. Negative change in CSA in jugular vein	0	1.4	1.9	4.3

CSA=cross-sectional area; MS=multiple sclerosis.

Valsalva-induced venous reflux (VIR) was never seen in the deep cerebral veins. However, VIR of the extracranial veins was present in 111 of 276 (40.2%) of the study participants. The proportion of subjects with any VIR in the extracranial veins was 75 of 206 (36.4%) and 36 of 70 (51.4%) in MS versus non-MS subjects (*p*=0.027, Pearson chi-square). Subgroup data with regard to VIR are available as Supplementary Table 2.

Table 4 illustrates the jugular vein CSA and venous velocities across MS versus non-MS subjects, right versus left side, and supine versus upright positions. No significant differences emerged between MS and non-MS subjects for extracranial or intracranial velocities. The CSA of the upright jugular vein was lower for MS versus non-MS subjects (0.17±0.14 vs 0.22±0.18cm^2^, *p*=0.03, not corrected for multiple comparisons).

**TABLE 4:** Jugular Vein CSAs and Ultrasound Velocities Compared across MS versus Non-MS Participants

Vein	Position		All MS Subjects				All Non-MS Subjects			
				
			Right		Left		Right		Left	
						
			No.	Mean±SD	No.	Mean±SD	No.	Mean±SD	No.	Mean±SD
Internal jugular CSA, cm^2^	Supine		205	0.80±0.03	206	0.60±0.03	70	0.84±0.06	70	0.67±0.05

	Upright		201	0.20±0.14	201	0.17±0.14^a^	69	0.22±0.16	67	0.22±0.18^a^

Internal jugular, cm/s	Supine	Max	204	60.40±36.41	204	50.85±30.44	70	64.73±38.22	70	52.05±29.19

		Min	204	21.35±18.09	204	16.82±14.60	70	19.35±19.59	70	16.23±17.41

	Upright	Max	199	75.80±46.71	198	57.95±41.37	68	75.63±38.51	66	50.14±33.16

		Min	199	52.63±36.71	198	37.05±32.15	68	50.55±30.59	66	31.88±27.93

Vertebral, cm/s	Supine	Max	190	29.86±19.68	175	28.43±18.09	64	28.41±20.63	59	28.34±23.95

		Min	190	13.73±13.57	175	11.17±9.26	64	11.36±12.27	59	10.00±16.58

	Upright	Max	190	45.43±27.01	185	42.44±28.79	60	42.50±24.26	57	47.61±33.32

		Min	190	33.29±21.09	185	30.02±22.00	60	32.92±20.85	57	35.89±28.32

Basal of Rosenthal, cm/s	Supine	Max	194	10.51±2.54	190	10.45±2.70	68	10.66±1.99	69	11.08±2.80

		Min	194	7.27±1.90	190	7.14±1.89	68	7.56±1.66	69	7.74±2.30

	Upright	Max	193	10.43±2.60	187	10.54±2.49	69	10.45±2.31	69	10.73±2.47

		Min	193	6.80±1.72	187	7.10±1.76	69	6.93±1.65	69	7.30±1.85

Galen, cm/s	Supine	Max	188	11.71±3.77			65	11.72±3.04		

		Min	188	8.05±2.72			65	8.23±2.22		

	Upright	Max	182	10.91±3.15			60	10.21±1.93		

		Min	182	7.17±2.19			60	6.66±1.44		

All 64 separate comparisons were not statistically significantly different between MS and non-MS subjects.

a *p*≤0.03, MS vs non-MS. If Bonferroni corrected, statistical significance would be considered *p*<0.0008.

CSA=cross-sectional area; Max=maximum; Min=minimum; MS=multiple sclerosis; SD=standard deviation.

## Discussion

We failed, as have many others, to find any association between the incidence of venous outflow patterns detectable by NS and MS. Moreover, abnormal ultrasound findings such as stenosis or reflux were quite infrequent across our entire study population. Nor did venous velocities differ between our MS and non-MS patients. These results are consistent with many of the studies performed subsequent to the original publication of Zamboni et al. Taken together, we conclude that the ultrasound findings described as CCSVI are neither common nor associated with MS.

Although we strictly followed the published Zamboni methodology for assessing intra- and extracranial venous reflux, we also collected VIR. Although common in the extracranial veins, no cases of intracranial reflux were found under standardized Valsalva in the deep cerebral veins. Therefore, it appears that even under Valsalva conditions of 40 mmHg, jugular or vertebral vein reflux does not seem to transmit intracranially to the deep cerebral veins. Thus, our results question the validity of the hypothesis that venous reflux in the brain contributes to the pathophysiology of MS.

Our rate of successful insonation of at least 1 deep cerebral vein (95.6%) is consistent with published rates of cerebral vein identification using transcranial Duplex technology. In a cohort of 130 volunteers, Stolz et al identified the veins of Rosenthal and Galen in 90.8% of subjects.^10^ Of the 12 participants in our study without temporal windows, 4 had 1 extracranial Zamboni criterion finding (3 MS and 1 non-MS). Presuming all 3 MS patients were to have intracranial reflux, and thus 2 criteria for CCSVI, the proportion of subjects with at least 2 criteria would be 5.34% for MS and 7.14% for non-MS (*p*=0.577, Pearson chi-square). In our experience, detection of the internal cerebral veins is challenging and only rarely insonated through the transtemporal bone window. Published detection rates are low and range from 13 to 34%.^10^,^11^ As a result, we focused our protocol on the veins of Galen and Rosenthal.

Many factors might explain the discrepant neurosonology results across available CCSVI publications. First, the ultrasound technique published by Zamboni and colleagues has been inconsistently described. For example, the definition of jugular vein stenosis was described as ≥50% CSA reduction in 1 article^2^ and ≤0.3cm^2^ in another.^1^ Second, the technique (size and placement) of spectral Doppler sample gates has not been described in detail. Third, major ultrasound societies and accrediting bodies require spectral Doppler to record duration for venous reflux testing.^12^,^13^ Zamboni and colleagues failed to demonstrate examples of spectral Doppler waveforms of intracranial reflux that would allow accurate and precise venous reflux duration timing. Instead, their publications include only suboptimal color Doppler ultrasound images that do not show reflux duration and can suffer from aliasing, which can result in misdiagnosis of reflux or even vessel misclassification (eg, vein vs artery vs artifact). Lastly, the intracranial vein assessment has undergone dramatic change over their publications. In 2008 and 2009, they used a traditional transtemporal approach to insonate the deep cerebral veins.^2^,^14^ However, their screening protocol published in 2011, did not mention the transtemporal approach.^15^ Rather, a nontraditional ultrasound window was recommended using a supracondylar window, where the probe is placed on the cheek at the level of the condylar process of the mandible. This window purportedly allows insonation of the superior and inferior petrosal sinuses never described in previous publications, in which only the deep cerebral veins (Galen, Rosenthal, and internal cerebral veins) were interrogated. Most recently, Zamboni and colleagues have retracted intracranial venous reflux (original criterion #2) as no longer recommended, but rather relegated to “potential additional criteria” for diagnosing CCSVI.^15^,^16^ This is perplexing, in that the more proximal the impairment in venous outflow from the brain, the less opportunity there is for maintaining normal intravascular flow characteristics through collateral drainage. In this regard, it is noteworthy that we never encountered intracranial reflux in the deep cerebral veins when an adequate transtemporal window was available, as was the case in 96% of our study population.

A new ultrasound technique, quality Doppler processing (QDP) is used by some for CCSVI reflux screening. QDP has not been validated against traditional duplex ultrasound and is only available on the Esaote-Biosound MyLab 25Gold device (Esaote North America, Indianapolis, IN). Fox et al compared traditional transcranial Doppler (TCD) ultrasound with QDP in 20 MS subjects undergoing CCSVI testing.^17^ Seven of the 20 were found to have intracranial reflux on QDP, but none demonstrated reflux using traditional TCD duplex imaging. Thus, inconsistent and nonstandard ultrasound methodology in combination with nonvalidated equipment have led to uncertain accuracy of the proposed criteria.^18^

Our study has limitations. First, although our local NS expertise is strong, the study remains a single-center experience. The pending published results of a large, multicenter investigation that is ongoing in Italy may prove useful in this regard.^19^ Second, our center lacked multiple trained sonographers and image interpreters, precluding the exploration of inter-rater reliability. However, in the course of this study, alternative neuroimaging results based on MRV and TLV were obtained blind to the NS interpretations and are being correlated to evaluate the veracity of the conclusions based on ultrasound alone that will be the subject of future publications. Third, although we did not test whether the neurosonologist remained blinded to the diagnostic grouping (ie, MS versus non-MS) at the conclusion of the ultrasound examination, all subjects were instructed not to discuss any medical history during the examination, and obvious neurologic deficits were present in both some of the MS and some of the non-MS patients. Fourth, our measurement technique of venous blood flow velocities did not allow an estimation of cerebral blood flow volume as described by others.^3^ Fifth, our measurement of B-mode jugular stenosis (Zamboni criterion #3) did not include assessment of venous anomalies (eg, vascular webs, dysfunctional valves), which were not described in the original articles,^1^,^2^ but later added to consensus protocols not available at the time we began our study.^15^,^16^ Therefore, we cannot comment on the prevalence of these anomalies or whether they exist to a greater degree in MS compared with non-MS subjects based on prospective and systematic evaluation by NS alone. Lastly, we did not ask participants to undergo repeated testing, so reproducibility of abnormal findings could not be assessed.

In conclusion, NS findings described as CCSVI were infrequent in our study participants and when found did not distinguish MS from other subjects. Our findings do not support the postulate that CCSVI is causally associated with MS. Moreover, based on our experience, the use of neurosonology as a screening tool for selecting CCSVI candidates for experimental interventional studies will yield few candidates with patterns consistent with postulated impairment of venous outflow.
