# Supplementary material for: Chronic Cerebrospinal Venous Insufficiency: Case–Control Neurosonography Results
Source: Ann Neurol. 2013 Feb 26;73(6):721–8. doi: 10.1002/ana.23839 (PMC3657573; doi:10.1002/ana.23839)
Supplement: Supplementary file 1 [file ana0073-0721-SD1.doc]

Supplementary Table 1. Demographic data of the participant subgroups at the time of ultrasound.

| **Diagnosis** | **Age in Years** | **% Female** | **Symptom Duration in Years** | **Duration of Diagnosis in**  **Years** | **EDSS** |
| --- | --- | --- | --- | --- | --- |
| Clinically Isolated Syndrome (12) | 52.24 ± 9.6^1^  55.6 (31.8 – 64.6)^2^ | 66.7 | 2.5 ± 2.1  2.0 (0.1 – 5.8) | - | 0.8 ± 0.9  0.5 (0 – 2) |
| Relapsing Remitting (128) | 45.8 ± 9.9  46.3 (21.3 – 65.9) | 73.4 | 12.7 ± 9.0  11.5 (0.2 – 39.0) | 8.5 ± 7.0  7.6 (0 – 27.3) | 1.8 ± 1.5  2.0 (0 – 6.5) |
| Secondary Progressive (48) | 51.1 ± 8.5  52.7 (30.9 – 65.1) | 66.7 | 20.3 ± 10.5  20.6 (4.6 – 50.9) | 14.3 ± 8.1  12.6 (0.9 – 36.1) | 4.5 ± 1.4  4.5 (2.0 – 7.0) |
| Primary Progressive (15) | 55.9 ± 8.0  58.6 (40.7 – 67.4) | 73.3 | 9.9 ± 7.9  7.7 (1.0 – 26.9) | 7.6 ± 7.8  4.0 (0.7 – 26.5) | 4.7 ± 1.9  4.5 (1.5 – 7) |
| Progressive Relapsing (3) | 54.3 ± 1.6  54.7 (52.9 – 55.6) | 66.7 | 16.8 ± 8.3  16.7 (8.6 – 25.1) | 10.7 ± 11.6  4.0 (0.4 – 23.3) | 3.2 ± 0.3  3.0 (3.0 – 3.5) |
| **All MS – (206)** | **48.3 ± 9.9**  **49.0 (21.8 – 67.4)** | **71.4** | **13.7 ± 10.0**  **(0.1 – 50.9)** | **9.9 ± 7.8**  **8.7 (0 – 36.1)** | **2.6 ± 2.0**  **2.0 (0 - 7)** |
| Healthy Volunteers (11) | 44.9 ± 14.1  45.1 (26.9 – 63.4) | 63.6 |  | | |
| Other Neurologic Diseases (37) | 41.7 ± 12.0  45.6 (18.4 – 60.0) | 70.3 |  | | |
| Stroke/TIA (22) | 48.4 ± 9.3  49.1 (29.9 – 65.0) | 54.5 |  | | |
| **All non-MS – (70)** | **44.3 ± 11.8**  **46.1 (18.4 – 65.0)** | **64.3** |  | | |

^1^Mean ± SD

^2^Median (range)

Supplementary Table 2. Valsava-induced venous reflux (VIR) in the extracranial vessels (jugular and vertebral veins) tabulated by participant category. No participants were found to have intracranial VIR in the deep cerebral veins.

| **Diagnosis** | **All Subjects** | **Subjects with Extracranial VIR** |
| --- | --- | --- |
| MS – Clinically Isolated Syndrome | 12 | 4 |
| MS – Relapsing Remitting | 128 | 43 |
| MS – Secondary Progressive | 48 | 18 |
| MS – Primary Progressive | 15 | 9 |
| MS – Progressive Relapsing | 3 | 1 |
| **Subtotal (All MS)** | **206** | 75 |
| Healthy Volunteers | 11 | 6 |
| Other Neurologic Diseases | 37 | 19 |
| Stroke/TIA | 22 | 11 |
| **Subtotal (All non-MS)** | **70** | 36 |
| All | 276 | 111 |
